# Could serum Vitamin-D be an indicator of the onset of membrane rupture?

**DOI:** 10.12669/pjms.41.2.8930

**Published:** 2025-02

**Authors:** Naziye Gurkan, Göksenin Unluguzel Ustun

**Affiliations:** 1 Naziye Gurkan, Department of Obstetrics and Gynecology, Medical Park Hospital, Samsun, Turkey; 2 Göksenin Unluguzel Ustun Department of Medical Biochemistry, Training and Research Hospital, Samsun, Turkey

**Keywords:** Amniotic fluid-NF-κB, Inflammation, Labor, Serum Vitamin-D

## Abstract

**Objective:**

Although Vitamin-D (VD) deficiency in pregnancy continues to be an important public health problem, the effects of VD on fetal outcomes are not clear. VD can show its effects at the feto-maternal junction via nuclear factor-kB (NF-kB). This study was planned to determine the effect of changes in serum VD levels on amniotic fluid NF-kB levels.

**Methods:**

Participants were selected among patients who applied to Samsun Medikalpark Hospital Gynecology and Obstetrics outpatient clinic between November 1, 2022 and November 1, 2023. Sixty patients whose serum VD measurements were performed at 24 weeks of gestation were included in the study. The pregnant women were divided into two equal groups according to their serum VD levels (n=30). The patients in Group-1 consisted of 30 patients with a VD level less than 20 ng/ml (VD deficienct group) and Group-2 consisted of 30 patients with a VD level higher than 20 ng/ml (VD sufficienct group). NF-kB levels were measured by ELISA in amniotic fluid samples taken during cesarean section from patients in both groups.

**Results:**

The amniotic fluid NF-kB concentration of the VD sufficient group was found to be significantly lower than the VD deficient group. The amniotic fluid NF-kB levels of the VD deficient group were approximately two times higher than the VD sufficient group (6.36±2.12 ng/mL vs. 3.95±1.49 ng/mL, p< 0.01). After adjusting for gestational age, fetal birth weight, and parity, significant associations were found between VD and amniotic fluid NF-kB. The mean gestational ages at the time of cesarean section were similar in both groups.

**Conclusion:**

Although VD insufficiency causes early inflammatory changes in fetal membranes by increasing amniotic fluid NF-kB levels, it does not lead to preterm delivery.

## INTRODUCTION

During pregnancy, a pregnancy-specific decrease may be detected in VD levels due to reasons such as inadequate and irregular nutrition, increased skin pigmentation, absorption defects, and geographic height.^1^ Therefore, premature birth, hypertension, low birth weight, increased inflammation frequency increases in VD deficiency.^2^,^3^ Normal levels of Vitamin-D before pregnancy positively affect pregnancy outcomes. Providing nutritional counseling before pregnancy has a positive effect on Vitamin-D levels.^4^ On the other hand, the side effects of VD use during pregnancy are not yet clear. Although some publications do not recommend routine use of VD during pregnancy^5^,^6^, according to the guideline recommendation published by Endocrine Society in 2011, the daily amount of Vitamin-D required during pregnancy and lactation is a minimum of 600 IU. However, to achieve Vitamin-D levels > 30 ng/mL, an intake of 1500-2000 IU may be necessary.^7^

Circulating VD levels cannot always be considered too clearly indicate the VD status of the pregnant woman. The fetus must meet its VD needs from the mother. The placenta allows the transmission of maternal VD to the fetus.^8^ Correlation of maternal blood VD levels with cord blood VD levels indicates the importance of VD supplementation.^9^ Since the fetus and its appendages cannot receive enough VD in VD deficiency, there will be deterioration in immunomodulatory functions in addition to calcium and phosphate metabolism.^10^,^11^ Stimulation of neoangiogenesis due to increased inflammation in VD deficiency leads to hypertension and immune dysfunction, leading to the emergence of obstetric pathologies.^12^,^13^

The recommended cut-off values for VD deficiency differ according to different associations.^7^,^14^ Endocrine Society accepts serum 25(OH)D level as <20 ng/mL as deficiency, VD values between 20-30ng/ml as insufficiency, and >30 ng/mL as adequate/replated. The United States Institute of Medicine, on the other hand, accepted a serum 25(OH)D level of more than 20 ng/mL as sufficient.^15^

It has been reported in a study that severe Vitamin-D deficiency with a 25(OH)D concentration below <30 nmol/L (or 12 ng/ml) dramatically increases the risk of excess mortality, infections, and many other diseases, and should be avoided whenever possible.^16^

Under physiological conditions, fetal membranes undergo inflammatory changes throughout pregnancy and are programmed to rupture at term. COX-2 and prostaglandin pathways in fetal membranes may also participate in the inflammatory process in the fetal and maternal compartments and initiate labor. The earlier emergence of inflammatory changes before parturition in VD deficiency paves the way for preterm birth and related complications. For these reasons, it is critical to diagnose VD deficiency during pregnancy and take precautions.

VD plays a role in the maintenance of immunomodulatory and anti-inflammatory reactions during pregnancy. VD regulates these effects through T and B lymphocytes.^11^ As the risk of VD deficiency increases due to the physiological conditions of pregnancy, there is a tendency towards systemic inflammatory reactions. Fetal membranes and amniotic fluid are the feto-maternal compartments most affected by inflammatory reactions. The response of the fetal membranes to the increasing inflammatory process is a decrease in their tensile strength and subsequent rupture. The reflection of inflammatory reactions, which are thought to occur in the membranes in VD deficiency, to the amniotic fluid has not been shown to date. Inflammation in fetal membranes can affect amniotic fluid by activating the nuclear Factor-kB (NF-kB) pathway. NF-kB is an inflammation marker that is bound and inhibited in the cell cytoplasm.^17^ If the amniotic fluid NF-kB pathway is activated in pregnant women with VD deficiency, amniotic fluid NF-kB levels must be increased.^18^ This study was planned to measure NF-kB levels in amniotic fluid samples collected at the time of cesarean section in term pregnant women with VD deficiency. Patients with normal serum VD levels were taken as the control group.

## METHODS

Participants were selected among patients who applied to Samsun Medikalpark Hospital Gynecology and Obstetrics Outpatient Clinic between November 1, 2022 and November 1, 2023. Sixty patients whose serum VD measurements were performed at 24 weeks of gestation were included in the study. All of patients were multiparous. Since the onset of labor would affect the results of the study, cases who had previously had a cesarean section were included in the study. Nulliparous patients, those who had a normal birth, and patients whose labor pains had started were excluded from the study. The pregnant women were divided into two equal groups according to their serum VD levels (n=30). The patients in Group-1 consisted of 30 patients with a VD level less than 20 ng/ml (VD deficient group) and Group-2 consisted of 30 patients with a VD level higher than 20 ng/ml (VD sufficient group). Serum VD levels were determined according to the IOM (Institute of Medicine). NF-kB levels were measured by ELISA in amniotic fluid samples taken during cesarean section from patients in both groups.

Gestational age was calculated according to the last menstrual period and the first recorded USG data. After the patients were informed about the study and their consent was obtained, the volunteers were included in the study.The sample size was calculated using the two tailed test to compare the VD levels in the normal and low groups. On the basis of 0.05 alpha and 0.80 power, the required sample size for each group was determined as 30.

### Ethical Approval:

Approval was obtained before the local ethics committee Ref. Approval No.: 2022/196. Dated: November 1^st^ 2022.

VD replacement was not applied to the VD deficient group. The patients were included in the routine pregnancy follow-up until the term. Patients who requested VD replacement, those who gave birth before term, and those who developed gestational diabetes, hypertension, and preeclampsia during follow-up, those with renal pathology, PPROM, placental insertion anomaly and Type-2 diabetes history, those with systemic inflammatory disease and those who used anti-inflammatory drugs in the last three months were excluded from the study. Women with a history of preterm births were not included in the study. All patients delivered by elective cesarean section. Amniotic fluid samples were collected with the help of an injector after the fetal membranes were cut. Approximately 10-15 cc of fluid was taken from each patient. Subsequently, the fetus and its appendages were delivered. Those with high blood or vernix caseosa content and meconium amniotic fluid after centrifugation were not included in the study.

### Amniotic fluid NF-κB analysis with ELISA:

After the frozen amniotic fluid samples of both groups were thawed, the samples were centrifuged at 4000 rpm for eight minutes. Phosphate buffering was done before centrifugation. Samples removed from blood, debris and vernix were treated with the human NF-kB ELISA kit and measurements were made. This kit can measure NF-kB levels in human biological fluids with great accuracy. Measurements were made in accordance with the information in the NF-kB ELISA kit catalog (FineTest®, Fine Biotech- Wuhan, China. Cat. No.: EH3422) and procedures were performed according to the manufacturer’s instructions. Amniotic fluid NF-kB results were given as ng/mL. The kit had measurement sensitivity in the range of 0.3-20 ng/mL. This sensitivity made the results reliable. The lowest NF-kB level measured by the kit was 0.07. The intra-assay and inter-assay coefficients of variation of the NF-kB ELISA kit were <6% and <5%, respectively.

### Statistical analysis:

All analyses were performed on IBM SPSS Statistics for Windows, Version 27.0 (IBM Corp., Armonk, NY, USA) and GraphPad Prism 8.0 software (GraphPad Software, San Diego, California, USA). Whether the data showed normal distribution or not was examined with the Shapiro–Wilk test. Continuous variables were analyzed using the Mann Whitney U test. Spearman’s correlation analysis was used to determine the correlation between amniotic fluid NF-kB levels and demographic parameters. Multivariate regression analysis with appropriate adjustment for confounders was performed. Confounders were selected on the basis of their association with the outcomes of interest. Multivariable logistic regression analysis was performed to determine NF-Kb independently associated with the Vitamin-D deficiency after adjusted with age, parity, gestational age and fetal birth weight. p<0.05 values were accepted as statistically significant results. Data are presented as mean ± SD. P<0.05 was considered statistically significant.

## RESULTS

Maternal, obstetric and other demographic data of both groups (VD deficient and VD sufficient) are presented in Table-I in detail. Maternal and gestational ages of the two groups were similar. There was no significant difference between the groups in terms of obstetric data. Caesarean section at term was successfully applied to all patients. No major complication was detected. Amniotic fluid samples were successfully collected from patients in both groups. There was no significant difference between the averages fetal birth weight of both groups.

**Table-I:** Demographic and laboratory characteristics of Vitamin-D deficiency and control groups.

	Group-1(n=30) Vitamin-D deficient (<20 ng/mL)	Group-2 (n=30) Vitamin-D sufficient (>20 ng/mL)	p-values
Age (years)	28.23±4.04	27.73±3.50	0.611
VD level (ng/mL)	14.21±2.99	26.03±4.30	<0.001
Gravidity	3 (2-4)	3 (2.75-4)	0.345
Parity	2 (1-2.25)	2 (1-3)	0.244
Gestational age (weeks)	38 (37-38.25)	38 (37-39)	0.418
Fetal birth weight (gr)	2994.10±250.35	3012.63±270.13	0.784
Amniotic fluid NF-kB (ng/mL)	6.36±2.12	3.95±1.49	<0.001

**Fig.1:**
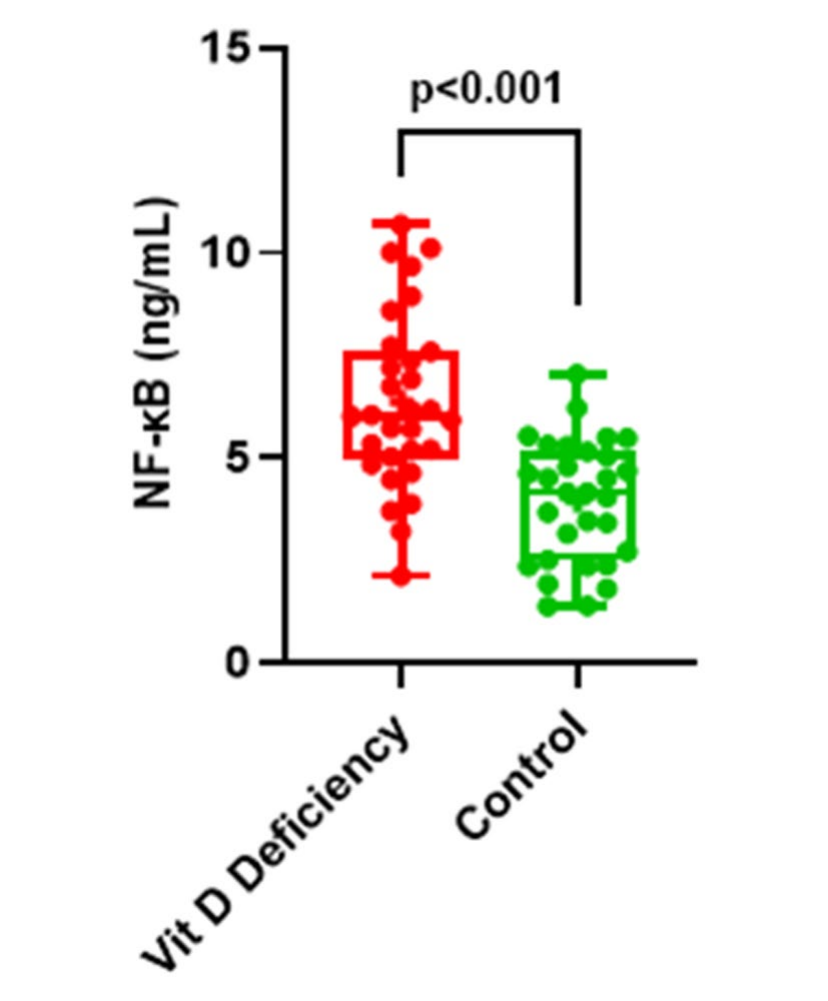
NF-kB levels of Vitamin-D deficiency and Vitamin-D sufficiency groups (control).

**Fig.2:**
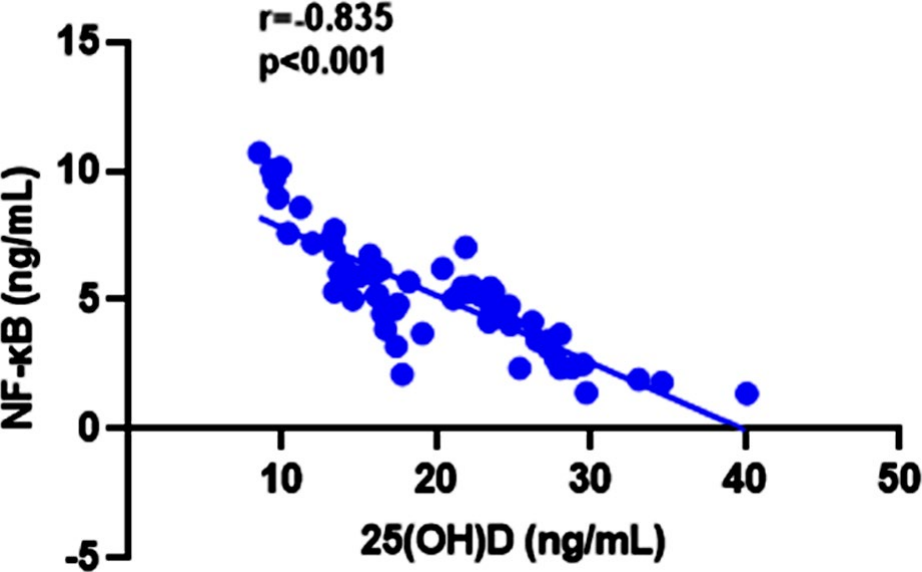
Graphical representation of the inverse relationship between amniotic fluid NF-kB and VD levels.

Data are given as mean ± standard deviation or median (25th and 75th percentile) for continuous variables according to normality of distribution as shown in Fig.1, the amniotic fluid NF-kB levels of the VD deficient group were approximately two times higher than the VD sufficient group (6.36±2.12 ng/mL vs. 3.95±1.49 ng/mL, p< 0.01). As shown in Table-II, a negative correlation was found between amniotic fluid NF-kB level and VD values of the VD deficient group (r=-0.835, p<0.001). As serum VD level decreased, amniotic fluid NF-kB levels increased (Fig.2).

**Table-II:** Correlation analysis between AF-NF-kB level and obstetrics parameters (n=60).

	r	p
Age (years)	0.726	<0.001
VD level (ng/mL)	-0.835	<0.001
Gravidity	-0.262	0.043
Parity	-0.167	0.202
Gestational age (weeks)	-0.037	0.779
Fetal birth weight (gr)	-0.049	0.710

In the VD sufficient group, there was no significant correlation between amniotic fluid NF-kB levels and serum VD values. There was no significant difference between VD values and other demographic and obstetric parameters for both groups. The mean gestational ages at the time of cesarean section were similar in both groups. VD deficiency did not lead to preterm delivery despite high NF-kB levels. The fetal weights of the VD-deficient group were similar to those of the VD sufficient group, suggesting that VD deficiency did not significantly affect fetal muscle and bone development. Multivariable logistic regression analysis had revealed that high AF-NF-kB (p<0.001) was independently associated with the Vitamin-D deficiency after adjusted with age, parity, gestational age and fetal birth weight (Table-III).

**Table III:** NF-kB measurements independently associated with the Vitamin-D deficiency, logistic regression analysis results (n=60).

	Unadjusted		Adjusted ^(1)^	

	OR (95% CI)	p	OR (95% CI)	p
NF-kB (ng/ml)	2.224 (1.425 – 3.473)	<0.001	7.323 (2.728 – 19.655)	<0.001

OR: Odds ratio, CI: Confidence interval. (1) Adjusted with age, parity, gestational age and fetal birth weight.

## DISCUSSION

If VD exhibits immunomodulatory and anti-inflammatory effects, amniotic fluid inflammatory markers will increase in VD deficiency. Since NF-kB is an important marker of the inflammatory pathway, we evaluated its relationship with serum VD levels in both patients with VD insufficiency and sufficiency.

Although recommended by the endocrine society, since VD replacement during pregnancy is not a mandatory part of antenatal care, it may be possible to detect changes in amniotic fluid in cases of VD deficiency. The reason why we chose amniotic sample for the study was that fetal membranes are one of the feto-maternal regions that are most and rapidly affected by inflammatory changes in pregnancy.

In the amniotic fluid evaluation, NF-kB levels of pregnant women with low VD levels were approximately two times higher than those with normal VD levels. This finding is evidence that the inflammatory pathway is activated in VD deficiency and the NF-kB-IkB complex is dissociated and NF-kB travels to the cell nucleus and stimulates the activation of inflammatory genes. Low NF-kB levels in pregnant women with normal VD levels are evidence that the NF-kB-IkB complex is not dissociated and the conditions for activation of the inflammatory pathway are not met. VD regulates its immunomodulatory and anti-inflammatory effects in the feto-placental region via NF-kB, VD receptors, natural killer cells, macrophages, and T lymphocytes.^19^,^20^ In experimental placental inflammation models, the NF-kB pathway is blocked by giving VD.^21^ The increase in NF-kB levels in the VD deficient group may be due to insufficient stimulation of VD receptors and activation of immunomodulatory cells. However, the increase in NF-kB levels in the group with decreased VD levels did not lead to premature rupture of membranes and early labor. This finding suggests that the inflammatory process in the fetal membranes alone is not sufficient to initiate labor. The fact that the birth times of both groups are similar suggests that the VD levels are not the only determinant in the rupture of the membranes and the onset of labor. In addition, studies have shown that the 1.25 (OH)2D3 receptor (VDR) and the one alpha hydroxylase enzyme that activates Vitamin-D are significantly expressed in the placenta and decidua and show autocrine and paracrine effects, thus VD may play a potential role in fetoplacental development 1.25 (OH)2D3 regulates key implantation-related target genes such as HOXA10 when bound to VDR (1.25 (OH)2D3 receptor), while its strong immunosuppressive effects may play a role in implantation tolerance.^22^,^23^

According to a study, thyroid stimulating hormone and blood glucose levels in pregnant women were negatively associated with VitD levels. Therefore, serum 25-OH-D level can be used as an important reference index for gestational diabetes mellitus with subclinical hypothyroidism, and maintaining it at normal level is of great clinical significance.^24^

A Cochrane meta-analysis evaluating data from the 2024 update found very uncertain evidence for preeclampsia, gestational diabetes, preterm birth, or nephritic syndrome when compared with Vitamin-D supplementation alone, no intervention, or placebo. All findings warrant further investigation. Additional rigorous, high-quality, and larger randomized trials are needed to evaluate the effects of Vitamin-D supplementation in pregnancy, particularly in relation to the risk of maternal adverse events.^25^,^26^

Indeed, at the onset of labor, the inflammation of the fetal membranes, the decrease of their tensile strength and their rupture are the chain of events that take place in the last stage. In the initial stage of labor, there is a joint interaction of the feto-maternal compartments, fetal adrenal cortex and maternal central nervous system. In the absence of central and adrenal axis activation, the increase in NF-kB in fetal membranes does not induce labor. The level of NF-kB to induce labor by decreasing the fetal membrane tensile strength is unknown. It is known that NF-kB reduces tensile strength in membranes by reducing intracellular adhesion molecules and matrix metalloproteinases.^22^ Since VD’s effect on NF-kB is regulated by the VD receptor, receptor expression defect may occur in VD deficiency. The increase in inflammatory molecule synthesis in VD receptor knockout animals suggests that VD blocks NF-kB through its own receptor. In fact, this condition may not have a clinical reflection since VD deficiency is balanced locally by local VD synthesis in the decidua and placenta.^22^,^27^ Studies measuring total VD, active VD, VD binding protein and local VD levels will be much more valuable in terms of evaluating fetomaternal effects.^28^

### Limitations:

The small number of patients is one of the limitations of the study. Our study is important as it is the first study to investigate amniotic fluid NFkB levels in pregnant women with VD deficiency. In addition, not being able to make a comparison between the groups that received and did not receive replacement therapy is an important limitation.

## CONCLUSION

Determining the relationship between NFkB, an inflammatory marker, and Vitamin-D in amniotic fluid, which is the most important part of the fetomaternal unit, is important in terms of shedding light on inflammatory processes in pregnancy. Many studies have shown that Vitamin-D, which is now considered a hormone rather than a vitamin, acts on the fetomaternal unit via VD receptors. According to this study, although VD insufficiency causes early inflammatory changes in fetal membranes by increasing amniotic fluid NF-kB levels, it does not lead to preterm delivery. However, since the fetomaternal effects of VD replacement are not clear, a clearer assessment can be made with studies analyzing more inflammatory markers in maternal serum, amniotic fluid and cord serum samples.

### Recommendations:

In future, studies measuring NFkB, especially in patients with VD replacement, are important in terms of showing the VD-NFkB relationship.
